# Ofatumumab in refractory anti-muscle-specific tyrosine kinase antibody-positive myasthenia gravis: a case report of successful salvage therapy and mechanistic insights

**DOI:** 10.3389/fimmu.2026.1792262

**Published:** 2026-06-10

**Authors:** Yikai Zhang, Chunxi Li, Guixian Chen, Haoyou Xu

**Affiliations:** 1 Guangzhou University of Chinese Medicine, Guangzhou, China; 2 Department of Neurology, The Second Affiliated Hospital of Guangzhou University of Chinese Medicine, Guangzhou, China

**Keywords:** B-cell depletion, case report, MuSK-MG, myasthenia gravis, ofatumumab, refractory case, salvage therapy

## Abstract

Muscle-specific tyrosine kinase antibody-positive myasthenia gravis (MuSK-MG), a subtype of myasthenia gravis (MG), follows an acute and severe disease course with suboptimal responses to standard treatments. Ofatumumab is a fully humanized anti-CD20 monoclonal antibody that induces potent and sustained B-cell depletion. Nonetheless, there are few reports on its use in sequential therapy failure after the failure of conventional drug treatment in MuSK-MG patients, especially in the Asian population. We report a 32-year-old female patient with anti-MuSK antibody-positive myasthenia gravis who was treated with ofatumumab as a rescue therapy after failing multiple treatments such as intravenous immunoglobulin (IVIG), plasma exchange, and efgartigimod, progressed to a crisis state necessitating prolonged mechanical ventilation. After the addition of ofatumumab to the combination of tacrolimus and corticosteroids, the clinical symptoms were significantly improved, the patient was successfully weaned from ventilator-assisted ventilation, the bulbar function was improved, and the limb muscle strength was significantly restored, and the patient was finally discharged uneventfully. This case is the first report of ofatumumab successfully treating refractory MuSK-MG in China, providing real-world evidence for its pivotal role in the treatment sequence. It also suggests that its characteristics, such as subcutaneous administration and low immunogenicity, may render it particularly suitable for high-risk patients, including those with concurrent infections.

## 1 Introduction

Myasthenia gravis (MG) is a rare autoimmune disorder of the neuromuscular junction, with a pathological core involving autoantibody-mediated synaptic transmission impairment and complement system activation (1, 2). The disease is closely associated with antibodies against specific proteins in the postsynaptic membrane. Among patients previously diagnosed as anti-acetylcholine receptor (AChR) antibody-negative MG, approximately 5% test positive for muscle-specific tyrosine kinase antibodies (MuSK-Abs) (3, 4). Among MG subtypes, MuSK-MG typically presents with preferential involvement of bulbar and respiratory muscles, a propensity to develop myasthenic crisis, and a considerably higher incidence of respiratory failure (5).

Standard MG management includes acetylcholinesterase inhibitors, corticosteroids, conventional immunosuppressants (e.g., azathioprine, mycophenolate mofetil), IVIG, and PLEX (6). For refractory cases, targeted biologics like the C5 complement inhibitor eculizumab and the FcRn antagonist efgartigimod alfa are also employed (7). Nonetheless, a subset of MuSK-MG patients responds poorly to these conventional treatments, exhibiting a refractory course that necessitates more effective biological targeted strategies.

The pathogenic antibodies in MuSK-MG are predominantly of the IgG4 subclass, which do not activate complement but instead directly interfere with the interaction between MuSK and LRP4, leading to impaired clustering of acetylcholine receptors (8, 9). This distinct mechanism explains the limited efficacy of complement-targeted therapies (e.g., eculizumab) in these patients. Consequently, strategies directly targeting B cells to reduce pathogenic antibody production at the source, such as anti-CD20 monoclonal antibodies, hold a clear rationale for MuSK-MG treatment (10).

Rituximab, an anti-CD20 antibody, has demonstrated efficacy in MuSK-MG. However, its murine-human chimeric structure can induce immunogenicity, leading to anti-drug antibody formation and potentially impacting long-term efficacy (11–13). Ofatumumab, a fully humanized anti-CD20 antibody, offers potential advantages in safety and convenience due to its lower immunogenicity, enhanced complement-dependent cytotoxicity (CDC), and subcutaneous route of administration (14). Beyond immunogenicity, the pharmacokinetic profiles of the two agents diverge notably. Rituximab is administered as a several-hour intravenous infusion. This dosing pattern is associated with high peak serum concentrations and pronounced inter-dose troughs, resulting in oscillatory B-cell depletion. In contrast, ofatumumab is approved for subcutaneous injection (20 mg given on days 0, 1, and 2, followed by monthly dosing starting from week 4), which achieves sustained steady-state exposure and more predictable B-cell depletion kinetics. This subcutaneous formulation enables patient self-administration, and may improve long-term disease control (15). Despite these theoretical advantages, clinical reports on ofatumumab for MuSK-MG, especially in multiply treatment-refractory cases, are extremely limited, and none exist within the Chinese population.

Here, we describe the first case of refractory MuSK-MG in a Chinese patient successfully treated with ofatumumab as salvage therapy, highlighting its potential advantages and clinical considerations.

## 2 Case presentation

A 32-year-old female patient presented with paroxysmal cough and expectoration in mid-December 2024, followed by progressively worsening dyspnea and dysphagia. Symptoms exhibited diurnal fluctuation, being milder in the morning and worsening towards the evening. Before the confirmation of MuSK antibody positivity, repetitive nerve stimulation (RNS) testing was performed and revealed a positive low-frequency decrement in the left orbicularis oris muscle. Due to the critical condition, the patient subsequently received intravenous immunoglobulin (IVIG) pulse therapy and plasma exchange. On January 19, 2025, the patient tested positive for MuSK-Ab [1:320, determined by cell-based assay (CBA)] at an external hospital and was diagnosed with Musk-MG. The patient received combined immunosuppressive therapy with pyridostigmine, mycophenolate mofetil, methylprednisolone, and tacrolimus. From December 30, 2024, to January 3, 2025, the patient received intravenous immunoglobulin (IVIG) pulse therapy (12.5 g daily for 5 days). From January 9 to January 17, 2025, plasma exchange was administered every other day for a total of 5 sessions. After the first plasma exchange, a transfusion allergic reaction occurred. Subsequent prophylactic medication during the next four sessions prevented further transfusion-related adverse events. Despite these interventions, the patient’s symptoms showed no significant improvement.

Due to progressive disease, the patient was transferred to our institution on February 5, 2025. Upon admission, her condition was critically ill and required endotracheal intubation for assisted ventilation. Neurological examination revealed incomplete eyelid closure bilaterally, limited abduction and upward gaze of the eyeballs, muscle strength of grade 4 in all limbs, and grade 1 strength in neck flexors. Repetitive nerve stimulation (RNS) demonstrated a decremental response during low-frequency stimulation, leading to a diagnosis of MuSK antibody-positive myasthenia gravis with myasthenic crisis (MGFA Type V). The patient presented with weak breath sounds in both lungs, elevated white blood cell count and infection/inflammatory markers, and chest CT findings of infectious lesions in the lower lobes of both lungs. Multiple sputum cultures yielded pathogenic bacteria, including Stenotrophomonas maltophilia, Acinetobacter baumannii, and Pseudomonas aeruginosa, which were consistent with the manifestations of pneumonia. The patient was sequentially treated with cefoperazone-sulbactam, tigecycline, piperacillin-tazobactam, ceftriaxone, and ceftazidime for anti-infective therapy. Throughout the course, the antibiotic regimen was dynamically escalated or de-escalated based on serial sputum culture results and inflammatory markers. Ultimately, ceftazidime was administered to cover Pseudomonas aeruginosa until the patient’s condition stabilized. In addition, the patient had hypoproteinemia, electrolyte disturbances, and PICC catheter-related thrombosis.

The patient had no relevant medical history, infectious diseases, or malignancies, nor any history of surgery, trauma, or relevant family history.

## 3 Therapeutic intervention and follow-up outcomes

After being transferred to our hospital, due to the presence of endotracheal intubation, the patient was discontinued from pyridostigmine. The combined immunosuppressive therapy was as follows: mycophenolate mofetil, methylprednisolone, and tacrolimus. Considering that FcRn blockade can reduce IgG levels of all subclasses, and given the patient’s critical condition with ventilator dependence, the research team attempted to use a rapidly acting agent, efgartigimod. Two doses of efgartigimod alfa (400 mg each, administered one week apart) were given for antibody clearance on February 8 and February 15, 2025, respectively. However, during this period, repeated attempts at weaning from mechanical ventilation were unsuccessful, arterial blood gas analysis persistently showed hypercapnia (PCO_2_ 45–72.8 mmHg), and no significant improvement in muscle strength was observed. In the absence of clinical improvement, the course of treatment was not completed. Given the lack of significant clinical improvement after multiple lines of immunotherapy including high-dose steroids, conventional immunosuppressants, IVIG, PLEX, and a short course of an FcRn antagonist, the patient’s condition was consistent with a refractory course of MG.

Given the poor response to multiple immunosuppressants and biological agents, alongside the presence of recurrent severe infections and ventilator dependency, the treatment team decided to initiate B-cell targeted therapy after careful consideration. Given the advantages of ofatumumab as a fully human monoclonal antibody—including lower immunogenicity and stronger complement-dependent cytotoxicity compared to first-generation anti-CD20 monoclonal antibodies (e.g., rituximab)—as well as the patient’s family’s preference for the subcutaneous route of administration, we commenced ofatumumab treatment on February 28, 2025 (subcutaneous injection of 20 mg at weeks 0 and 1, followed by monthly subcutaneous injections of 20 mg starting from week 2). The patient tolerated the first dose well, with no significant adverse reactions observed. During the treatment period, non-invasive ventilator-assisted ventilation, anti-infective therapy, and nutritional support were continued concurrently.

**Figure 1:**
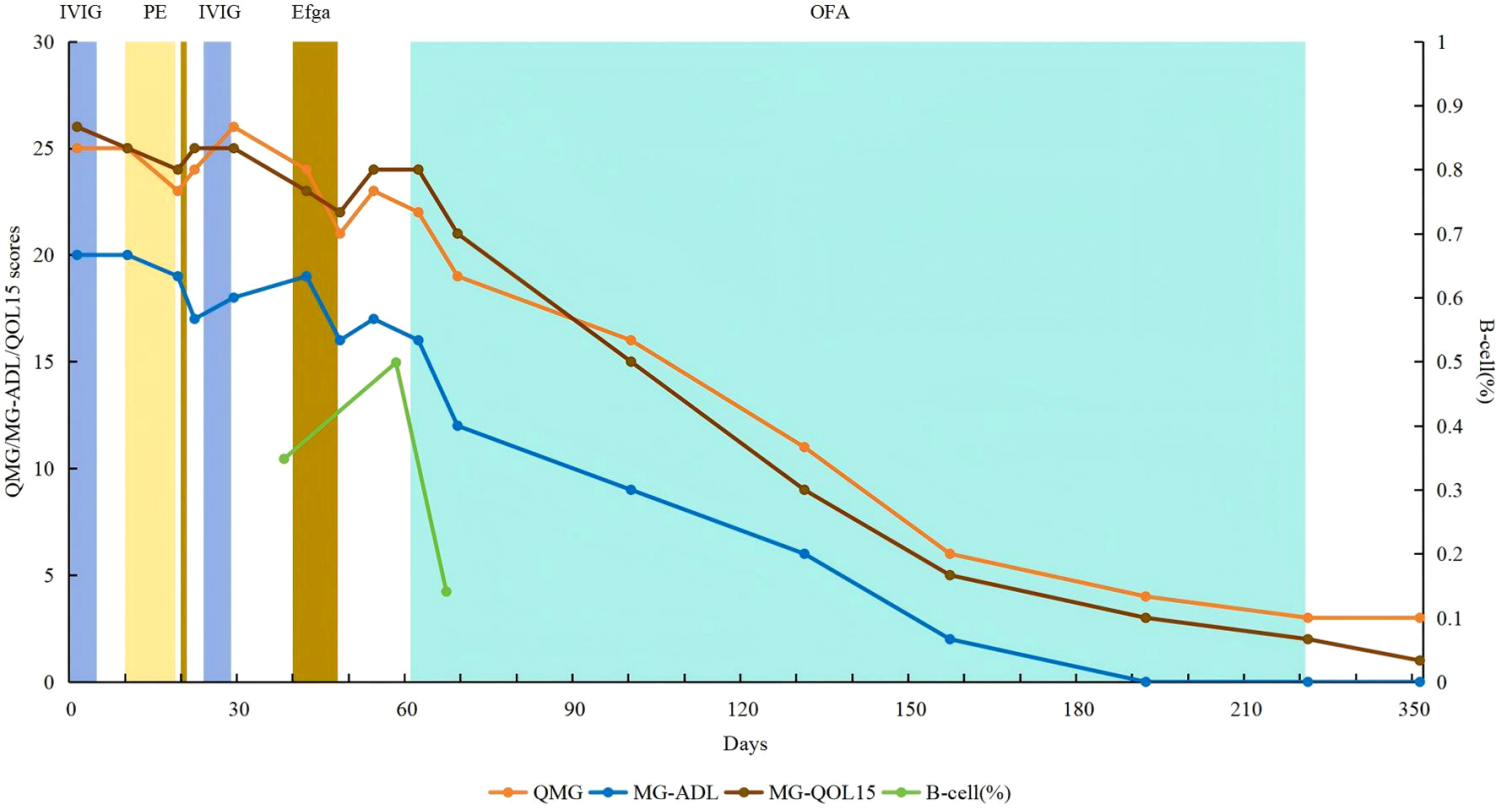
Evolution of QMG scores, MG-ADL scores, and B-cell (%) levels in our patient. Day 0 represents the day of hospital admission. QMG, Quantified Myasthenia Gravis; MG-ADL, Myasthenia Gravis Activities of Daily Living; MG-QOL15, Myasthenia Gravis Quality of Life 15-Item Scale; B-cell (%), B-lymphocyte percentage; IVIG, Intravenous Immunoglobulin; PE, Therapeutic Plasma Exchange; Efga, Efgartigimod; OFA, Ofatumumab.

During treatment with ofatumumab, the patient’s muscle strength gradually improved. Neck flexion strength recovered from grade 1 before treatment to grade 4, and incomplete eyelid closure improved. Sputum production decreased, and respiratory function steadily recovered. The patient was successfully weaned from invasive mechanical ventilation on March 2, 2025, and transitioned to non-invasive ventilation support; on March 6, 2025, non-invasive ventilation was completely discontinued, and the patient achieved stable spontaneous breathing. The patient’s Quantitative Myasthenia Gravis (QMG) and Myasthenia Gravis Activities of Daily Living (MG-ADL) scores also decreased significantly (**Figure 1**). Arterial blood gas analysis showed a reduction in partial pressure of carbon dioxide and improved oxygenation parameters. Peripheral blood lymphocyte subset analysis demonstrated a rapid decline in the proportion of B cells (CD19+), decreasing from 14.96% on February 26 to 4.23% on March 7. Throughout the entire treatment period, the patient’s complete blood count, albumin, lipid profile, liver, and kidney function remained within normal ranges, with no new infectious occurring. By the time of discharge on March 8, 2025, the patient had regained the ability to cough up sputum independently and eat orally, with significant recovery of limb muscle strength, achieving self-care in daily living activities. Post-discharge, the patient received monthly ofatumumab therapy (20 mg per dose, once monthly for 5 doses). Follow-up until December 2025 showed sustained improvement symptoms, with no choking during eating and complete independence in daily living activities. Scores on the QMG, MG-ADL, and QOL15 questionnaires continued to decline, as detailed in **Figure 1**. The patient’s weight increased from 33 kg at admission to 48 kg, with BMI restored to the normal range. Follow-up MuSK antibody titer: 1:30. The patient’s immunosuppressive regimen and treatment timeline are detailed in **Table 1**.

**Table 1:** Detailed immunosuppressive regimen and timeline.

Drug	Dosage	Start date	End date	Duration	Biological effect/notes
Pyridostigmine	60 mg q8h	2025-01-08	2025-02-05 (discontinued after transfer)	28 days	Partial symptom relief; discontinued due to risk of increased secretions
Mycophenolate mofetil	1.5 g/day	2025-01-15	2025-02-08	24 days	Immunotherapy strategy adjustment and escalation
Methylprednisolone	16 mg/day	2025-02-08	Ongoing	–	Dose escalation
Tacrolimus	3 mg/day	2025-02-08	Ongoing	–	Trough level 5–8 ng/mL
IVIG	0.4 g/kg total over 5 days	2024-12-30; 2025-01-23	2025-01-03; 2025-01-27	2 cycles of 5-day treatment each	Completed, no clinical response
Plasma exchange	5 sessions (qod)	2025-01-09	2025-01-17	5 sessions	Completed, allergic reaction after first session
Efgartigimod alfa	400 mg IV weekly	2025-01-19, 2025-02-08	2025-01-31, 2025-02-15	4 doses (cycle incomplete)	No clinical improvement (MG-ADL unchanged); stopped
Ofatumumab	20 mg SC (weeks 0,1, then monthly)	2025-02-28	Ongoing	–	–

IVIG, intravenous immunoglobulin. Q8h, quaque *8 hora*, every 8 hours. Qod, quaque *altera die*, every other day.

## 4 Discussion

MG is an autoimmune disorder characterized by impaired neuromuscular junction transmission mediated by autoantibodies. Among global MG patients, approximately 3-7% test positive for anti-MuSK antibodies, while this proportion may reach up to 40% in AChR antibody-negative MG patients (16). Unlike the common AChR antibody-mediated MG, MuSK-MG exhibits a distinct pathophysiology mechanism: its pathogenic antibodies are predominantly of the IgG4 subtype. Through Fab arm exchange, they form functional monovalent antibodies that specifically bind to the Ig1 domain of MuSK. This binding blocks MuSK-LRP4 interaction, inhibits MuSK phosphorylation, disrupts the Agrin-LRP4-MuSK-Dok7 signaling pathway, and ultimately impairs acetylcholine receptor aggregation (17). This mechanism determines the clinical features of MuSK-MG—predominant involvement of bulbar muscles, rapid disease progression, susceptibility to crises, and poor response to conventional acetylcholinesterase inhibitors (18).

Regarding treatment strategies, traditional therapeutic drugs such as glucocorticoids and nonsteroidal immunosuppressants demonstrate limited efficacy in MuSK-MG, accompanied by issues of poor tolerability and safety concerns (19, 20). Current systematic reviews, retrospective/prospective studies demonstrate that rituximab exhibits favorable efficacy in MuSK-MG, significantly improving scores on the MGFA grading system, MG-ADL, and QMG. It substantially reduces glucocorticoid dosage, with some patients achieving long-term remission or discontinuation of corticosteroids (11–13). The theoretical mechanism of its advantage may stem from the fact that pathogenic IgG4 antibodies in MuSK-positive MG are primarily produced by short-lived plasmablasts, which predominantly originate from CD20+ B cells. B-cell depletion therapy (e.g., rituximab) effectively eliminates CD20+ B cells and their precursors, blocking the continuous production of pathogenic IgG4 antibodies. This leads to a rapid decline in antibody titers and significant clinical improvement (10). However, the immunogenicity associated with its murine–human chimeric structure poses a major challenge to the long-term efficacy of rituximab. A retrospective study of 101 MG patients reported that 37.6% developed anti-drug antibodies (ADAs) following rituximab treatment (21). This may accelerate drug clearance and lead to diminished clinical response.

In contrast, ofatumumab, as a fully humanized monoclonal antibody, exhibits lower immunogenicity with a lower incidence of ADA formation compared to other non-fully human monoclonal antibodies (22). Neutralizing antibodies were rarely observed in multiple sclerosis studies. This provides assurance of sustained efficacy for refractory patients requiring long-term, repeated dosing to maintain disease control. Beyond immunogenicity advantage, ofatumumab also demonstrates potential theoretical superiority in its mechanism of action. Ofatumumab binds two distinct epitopes on the CD20 molecule (the large and small loops), whereas rituximab binds only the large loop epitope (23). This binding difference confers higher affinity of ofatumumab for CD20, enabling more efficient B-cell lysis (24), including partially resistant to rituximab elimination. Additionally, ofatumumab exhibits stronger complement-dependent cytotoxicity (CDC) activity than rituximab. Preclinical data indicate its CDC activity is approximately tenfold that of rituximab (25), enabling more effective induction of B-cell lysis. In MuSK-MG, pathogenic IgG4 is primarily produced by short-lived plasma cells. Theoretically, enhanced B-cell clearance capacity facilitates faster and more complete reduction of pathogenic antibody levels (12, 17). Regarding administration routes, rituximab is delivered via intravenous infusion, often accompanied by infusion-related reactions requiring premedication management. In contrast, ofatumumab’s subcutaneous administration at low doses results in fewer side effects. For this critically ill patient with concurrent infection, selecting a treatment regimen that minimizes infusion reactions and borders on the acceptable threshold holds significant practical importance.

Moreover, ofatumumab’s mechanism extends beyond potent B-cell cytotoxicity and autoimmune antibody reduction to involve profound reprogramming of the immune regulatory network. In AChR-MG patients, treatment with ofatumumab restored circulating Tfh and Th17 cell counts to normal levels and rebalanced the Th17/Treg ratio, providing a plausible mechanistic framework for its efficacy in MuSK-MG (26). By eliminating this central B-cell hub, ofatumumab may also disrupt the T-cell help essential for maintaining autoantibody production and suppress a broader inflammatory environment, thereby achieving rapid clinical improvement and stability. In summary, our selection of ofatumumab over rituximab was based on a thorough weighing of drug characteristics against the patient’s individual circumstances.

Several factors contributed to the delay in initiating anti-CD20 therapy earlier in this patient’s disease course. For one thing, in China, rituximab and other B-cell-depleting agents have not yet obtained an official indication for the treatment of myasthenia gravis; their use remains off-label and is often limited by reimbursement policies or regulatory restrictions. As a result, anti-CD20 therapy tends to be held back as a second- or third-line option, considered only after conventional immunosuppressants like glucocorticoids, azathioprine, or mycophenolate mofetil have failed. Another critical consideration is that the patient was critically ill upon admission with a severe concurrent infection. The approved subcutaneous formulation of rituximab requires a fixed dose of 1,400 mg (co−formulated with hyaluronidase), which is substantially higher than the cumulative dose of ofatumumab (20 mg monthly). Under these circumstances, early escalation to B−cell depletion therapy would raise legitimate concerns about further immunosuppression and exacerbation of the infection. Taken together, these practical realities explain why, despite emerging evidence supporting early B-cell depletion in MuSK-MG, a combination of healthcare-system, regulatory, and bedside decision-making barriers continues to hinder the timely application of this potentially disease-modifying therapy.

This report documents the first successful treatment of refractory MuSK-MG with ofatumumab in a Chinese patient, incorporating pre- and post-treatment MuSK antibody titers (1:320 → 1:30), longitudinal B-cell monitoring data, detailed clinical scores, and the use of contemporaneous medical records to reduce recall bias. The success of this case signifies a paradigm shift in therapy: For refractory MuSK-MG, the treatment focus should transition from mere antibody clearance or nonspecific immunosuppression to achieving comprehensive B-cell depletion and immune network reset.

More importantly, this case illustrates ofatumumab’s suitability for high-risk patients with severe infections: its subcutaneous administration avoids infusion-related reactions, eliminates complex premedication protocols, and reduces treatment complexity and risks. The successful treatment in this case also prompts forward-looking consideration: Could early application of B-cell depletion therapy (such as ofatumumab) in MuSK-MG patients, rather than during the salvage phase, more effectively curb disease progression and prevent critical episodes requiring mechanical ventilation? Given MuSK-MG’s rapid progression, early intervention may alter the disease course.

Naturally, this study is a single case report with inherent limitations, including the lack of more frequent monitoring of MuSK antibody titers and the inability to clearly distinguish the contribution of each drug in the combination therapy. Although the “carry-over” effect of previous treatments still introduces a certain degree of confounding, this association strongly supports the therapeutic effect of ofatumumab. The follow-up duration in this study was 12 months, and the long-term durability beyond 24 months remains not fully determined.

Furthermore, the proposed direct effect of ofatumumab on a presumed CD20+ pathogenic T-cell subset, suggested in some studies, remains speculative in this patient due to the absence of specific immunophenotypic analysis. Nevertheless, the strong temporal correlation between clinical recovery and B-cell depletion strongly suggests a potential core therapeutic mechanism for ofatumumab. Future prospective studies and larger registrational trials are needed to validate these findings, further explore the immunorestoration kinetics of B-cell depletion therapies in such patients, investigate the association between antibody dynamics and clinical outcomes, and assess the synergistic potential and optimal timing of combination therapies with other targeted agents such as FcRn antagonists and complement inhibitors. This will advance the development of personalized, multi-targeted treatment strategies.

## 5 Conclusion

Refractory MuSK-MG remains a clinical challenge. Its rapid progression, prominent bulbar involvement, and poor response to conventional therapies underscore the urgent need for novel effective treatment options. This case demonstrates that for refractory MuSK-MG unresponsive to multiple therapies, ofatumumab—a fully human anti-CD20 monoclonal antibody—can effectively deplete B cells in a durable and low-immunogenic manner. It remains a critical intervention even during mid-to-late disease progression, exhibiting favorable tolerability and safety. Ofatumumab may serve as an effective rescue therapy option for refractory MuSK-MG patients after failure of traditional therapies. While ofatumumab can be effective even in late-stage refractory disease, the optimal timing of B-cell depletion therapy in MuSK-MG is likely early in the disease course. Combination therapy with other immunosuppressants requires careful individualization, and the role of ofatumumab as part of a multi-agent regimen should be investigated in future prospective studies. Future studies should focus on defining its optimal therapeutic window and conducting longitudinal monitoring MuSK antibody titers along with immune cell dynamics, to advance precision immunotherapy for refractory MG.

## Patient perspective

The patient described her battle with MuSK-MG as an exceptionally arduous and uncertain challenge. The rapidly progressive dyspnea and dysphagia, the ineffectiveness of multiple treatment regimens, and the distress caused by invasive interventions such as endotracheal intubation left the patient feeling profoundly helpless and anxious. After thorough discussion, the patient consented to receive ofatumumab, a relatively novel therapeutic option. Fortunately, following the initiation of treatment, the patient’s condition improved markedly, with gradual recovery of muscle strength, normalized breathing, and regained ability to perform basic daily activities. Ultimately, the patient was successfully weaned off mechanical ventilation and discharged from the hospital. Currently, the patient’s quality of life has improved significantly, accompanied by restored body weight and renewed confidence in the future. Informed consent has been obtained from the patient, who has expressed satisfaction with the treatment received.

## Data Availability

The original contributions presented in the study are included in the article/supplementary material. Further inquiries can be directed to the corresponding author.
